# Autoimmune hepatitis presenting as severe anemia

**DOI:** 10.1002/jpr3.12076

**Published:** 2024-04-29

**Authors:** Brandon J. Calley, Alexandra Polovneff, Kathryn Henry, Paula North, David C. Moe, Cara L. Mack

**Affiliations:** ^1^ Medical College of Wisconsin Milwaukee Wisconsin USA; ^2^ Department of Pathology Medical College of Wisconsin Milwaukee Wisconsin USA; ^3^ Department of Radiology, Division of Pediatric Radiology Medical College of Wisconsin Milwaukee Wisconsin USA; ^4^ Department of Pediatrics, Division of Pediatric Gastroenterology, Hepatology and Nutrition Medical College of Wisconsin Milwaukee Wisconsin USA

**Keywords:** autoimmune hemolytic anemia, liver failure, portal hypertension, splenorenal shunt

## Abstract

Autoimmune hepatitis (AIH) is relatively rare in children. Herein, our case demonstrates a unique presentation of AIH in a previously healthy 18‐year‐old female presenting with a mild cough, fatigue, and severe anemia (hemoglobin 2.9 g/dL). Initial evaluation revealed jaundice and scleral icterus, prompting transfer of care and further testing, which demonstrated severe microcytic anemia, pancytopenia, elevated liver enzymes, direct hyperbilirubinemia, and marked splenomegaly. Concern for autoimmune hemolytic anemia resulted in a delayed diagnosis. The combination of triple antibody positivity (anti‐nuclear antibodies, anti‐actin, and anti‐liver‐kidney microsomal‐1) and liver histology findings confirmed the diagnosis of AIH. Intravenous methylprednisolone was initiated to induce remission. Due to pancytopenia and persistently elevated international normalized ratio, tacrolimus was chosen as the maintenance immunosuppression instead of azathioprine. This case highlights several significant considerations for clinicians, including the importance of a timely clinicopathologic diagnosis, the severe anemia presentation secondary to hypersplenism, and the rare finding of triple autoantibody‐positive AIH.

**Abbreviations**
AIH: autoimmune hemolytic anemia
ANA: antinuclear antibody
CT: computed tomography
DNA: deoxyribonucleic acid
EGD: esophagogastroduodenoscopy
IgG: immunoglobulin G
INR: international normalized ratio
IV: intravenous
LKM: liver‐kidney‐microsomal
PT: prothrombin time

## 1 INTRODUCTION

Autoimmune hepatitis (AIH) can present at any age with jaundice, fatigue, abdominal pain, arthralgia, and/or pruritus.^1^ Differentiation of the two types of AIH is based on the presence of specific autoantibodies.^1^ This case highlights triple autoantibody‐positive AIH presenting as severe anemia.

## 2 CASE REPORT

An 18‐year‐old previously healthy female presented with a 2‐week history of non‐productive cough and 2 days of mild fatigue. Other reviews of systems were negative. Their family history included essential thrombocythemia in her father. Detection of jaundice and scleral icterus at the urgent care facility prompted a referral to an outside emergency department. Laboratory evaluation revealed severe anemia (hemoglobin (Hb) 2.9 g/dL), leukopenia, thrombocytopenia, elevated total and direct bilirubin, elevated liver transaminases, and a positive direct Coombs (immunoglobulin ([IgG])) test (Table 1). Other laboratories included an elevated reticulocyte count (3.9%; normal range 0.8%−2.2%), decreased haptoglobin (24 mg/dL; normal range 35−250), elevated iron (537 mcg/dL), and a positive warm agglutinin assay. The peripheral blood smear was negative for spherocytes, schistocytes, and blasts. Physical examination demonstrated tachycardia and splenomegaly 13 cm below the left costal margin. Abdominal ultrasound revealed a nodular liver and splenomegaly. A hematology consult at the outside facility favored a presumptive diagnosis of autoimmune hemolytic anemia based on the positive Coombs test and the severity of the anemia. The patient was given a dose of IV methylprednisolone 40 mg, 2 units of packed red blood cells (pRBC), and transferred to our children's hospital under the general hospitalist service.

**Table 1:** Laboratory results throughout hospital admission and after discharge.

	Hb (g/dL)	WBC (10^3^/uL)	Platelets (10^3^/uL)	AST (IU/L)	ALT (IU/L)	Total/conjugated bilirubin (mg/mL)	PT (s)	INR	Total IgG (mg/dL)	ANA titer anti‐actin (U/mL) anti‐LKM (U/mL)
Reference ranges	12.5−16	4−10.5	150−450	5−35	10−35	0−1.1	12.4−14.6	<1	508−1080	<1:80 < 20 U/mL < 25 U/mL
Pre‐admit	2.9	2.7	102	247	135	6.8/2.5	**‐**	**‐**		
Day 1	6.1, 6.4, 6.9	3.1, 3.1, 6.5	115, 109, 106	238	156	7.5	23, 23.8	1.89, 1.97		>1:2560 27 57.2
Day 2	6.6	6.6	116	127	120	8.3/1.4	23.4	1.93		
Day 3	6.3	4.2	107	148	121	7.2/1.1	23.1	1.9		
Day 4	7.2	3.5	104	169	123	7.4/1.3	21.8	1.76		
Day 5	7.1, 8.1, 7.9, 9.5	3, 2.9, 2.7	87, 89, 91	218	130	6.7/0.7	23.8, 22	1.97	2240	
Day 6	8.8	4.4	100	179	123	6.5	20.8	1.66		
Day 7	8.4	6.1	91	122	106	4.5	21.9	1.77		
Day 8	‐	‐	‐	95	93	3.7	22.5	1.83		
Day 9	8.5	4.7	81	80	89	3.8	21.4	1.72		
Day 10	‐	‐	‐	71	89	3.3	21.6	1.74		
Day 11	8.5	3	80	61	81	3.1	22.4	1.82		
Day 12	‐	‐	‐	56	78	3.2/0	20.6	1.64		
2 months after discharge	11.1	3.9	81	47	39	1.1	‐	1.21		
4 months after discharge	12.1	3.9	95	58	40	1.5	‐	1.27	1451	

*Note*: Values separated by a comma indicate multiple sequential levels obtained on the same day.

Abbreviations: ALT, alanine aminotransferase; ANA, anti‐nuclear antibody; AST, aspartate aminotransferase; Hb, hemoglobin; INR, international normalized ratio; LKM, anti‐liver kidney microsomal antibody; PT, prothrombin time; WBC, white blood cell count.

**Figure 1:**
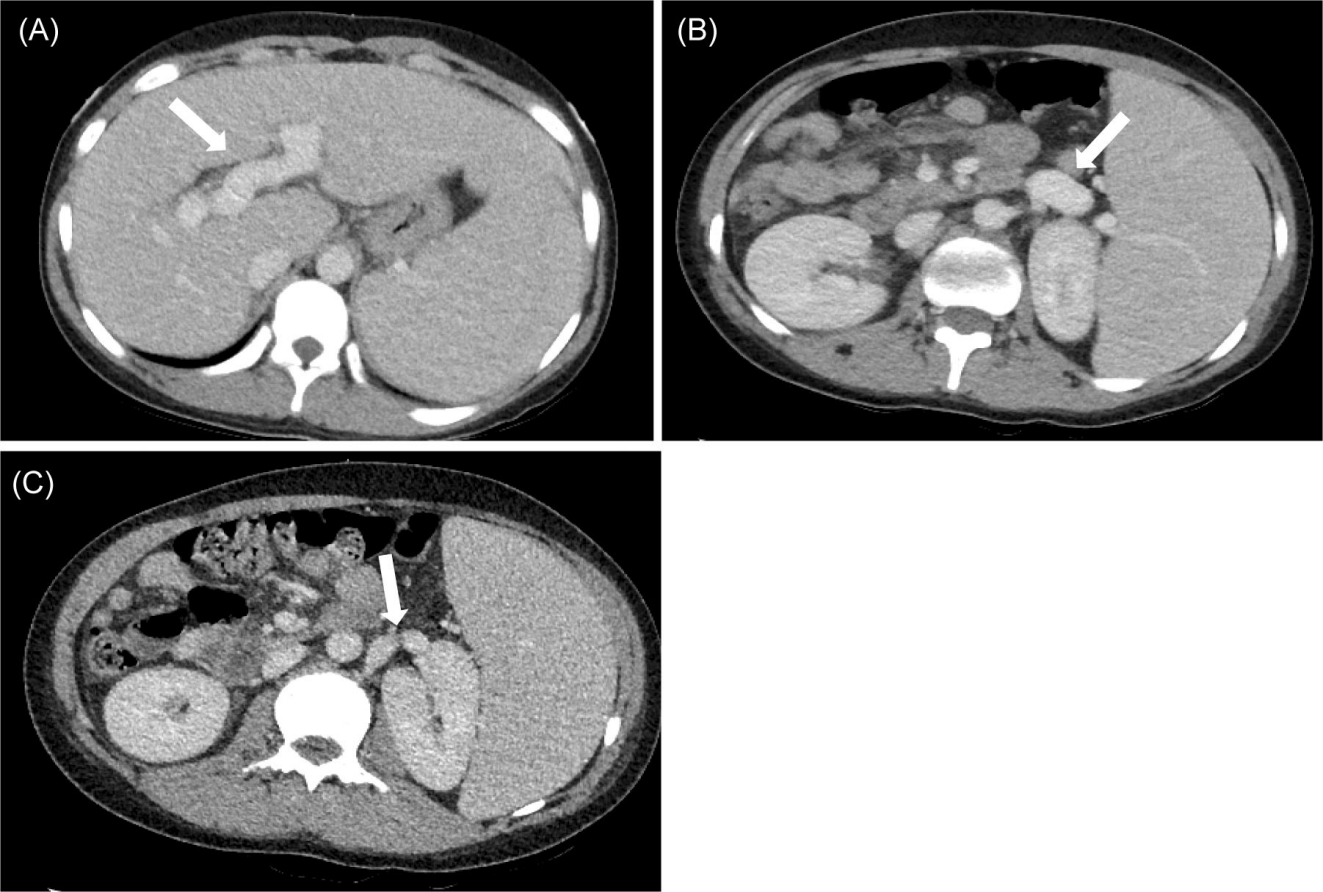
Abdominal CT scan findings of portal hypertension. (A) Axial CT with IV contrast demonstrates enlarged intrahepatic portal vein (arrow) with splenomegaly; (B) large splenic hilum varix (arrow); (C) varix drains to left renal vein as a splenorenal shunt (arrow). CT, computed tomography; IV, intravenous.

On admission, the hemoglobin improved to 6.1 g/dL after the pRBC transfusion. The hospitalist team obtained a chest and abdominal computed tomography scan that showed dilated portal and splenic veins, evidence of splenorenal venous shunt, and multifocal ground glass nodular opacities in the right upper lung (Figure 1). The hospitalist service ordered multiple consults, including oncology (concluded low likelihood for oncologic process due to lack of a mediastinal mass, lymphadenopathy, or blast cells); hematology (concluded low suspicion for autoimmune hemolytic anemia based on a repeat negative direct Coombs test [IgG and complement], normal lactate dehydrogenase [224 U/L], and the fact that there was no difficulty crossmatching the patient's red blood cells (RBCs)); and rheumatology (initial concern for Evans syndrome (ES), although less likely due to lack of evidence for autoimmune hemolytic anemia). Pulmonology and infectious disease consultations revealed evidence of nonnovel coronavirus infection but otherwise negative workup for multiple bacterial, viral, or atypical fungal infections. This included non‐detection of Hepatitis A−C, cytomegalovirus, and Epstein−Barr virus (polymerase chain reaction and IgM). Hepatitis E was not evaluated.

**Figure 2:**
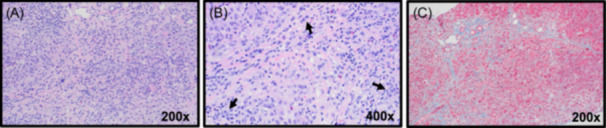
Liver histology of autoimmune hepatitis. Shown is the histologic confirmation of AIH. (A) H&E staining displays interface hepatitis. Lymphoplasmacytic portal tract infiltrates extend beyond the limiting plate into the parenchyma; (B) magnification of infiltrates: includes lymphocytes, neutrophils, eosinophils, and clusters of plasma cells (arrows); (C) trichrome stain (blue) showing portal tract bridging fibrosis. AIH, autoimmune hepatitis; H&E, hematoxylin and eosin.

Hepatology was then consulted, and the initial assessment was a chronic liver disease with portal hypertension and risk for developing acute or chronic liver failure (based on international normalized ratio [INR] approaching 2.0). Intravenous vitamin K (5 mg) was administered the first 3 days of admission, with minimal/no improvement in INR (see Table 1). The initial differential diagnosis included AIH, Wilson disease, alpha‐1‐antitrypsin deficiency, or primary sclerosing cholangitis. There was no history of hematemesis, melena, or hematochezia, and the stool guaiac test was negative. However, due to evidence of portal hypertension and the severe anemia presentation, an EGD was performed and showed grade 1 esophageal varices and mild portal hypertensive gastropathy, without evidence of recent bleeding. Laboratory results included elevated total IgG (2240 mg/dL) and positivity for all three autoantibodies: anti‐actin antibodies (27 U/mL; weakly positive 20–30 U/mL), anti‐liver‐kidney‐microsomal‐1 (LKM‐1) antibodies (57.2 U/mL; positive >25 U/mL), and anti‐nuclear antibodies (ANA) (>1:2560; positive ≥ 1:80). Negative findings for anti‐double‐stranded DNA, ceruloplasmin (normal), 24 h urine copper, and alpha‐1‐antitrypsin. Liver histology revealed a lymphoplasmacytic interface hepatitis and stage 2−3 fibrosis (Figure 2), confirming the diagnosis of AIH. Daily IV methylprednisolone was administered on hospital Days 5−11, followed by 40 mg daily of oral prednisone, with improvement in transaminases and INR (Table 1). In the setting of pancytopenia, there was concern that using azathioprine for maintenance therapy could worsen the pancytopenia. Therefore, the second‐line therapy chosen was tacrolimus, initiated on day 10 at ~0.05 mg/kg/dose twice daily, with goal troughs of 8−10. On hospital Day 12, the patient's INR decreased to 1.64, and liver enzymes and bilirubin continued to a downtrend; therefore, the patient was discharged to home on prednisone and tacrolimus. The prednisone was slowly weaned and discontinued by month 9 based on biochemical remission, and the patient remains on tacrolimus monotherapy.

## 3 DISCUSSION

This case presentation has several unique features. First, there was a delay in consulting hepatology and, therefore, a delay in obtaining the correct diagnosis and therapy. The delay was due in part to the initial concern for autoimmune hemolytic anemia (prompting hematology and rheumatology consults). In addition, there was an initial concern raised for ES due to the presentation of pancytopenia. A diagnosis of exclusion, ES typically involves depletion of two or more cell lines, autoimmune hemolytic anemia, and immune thrombocytopenia.^2^ The association of AIH and ES is rare, especially among the pediatric population; however, it has been previously reported.^3^

Second, the severe anemia presentation (Hb 2.6 g/dL) in AIH is highly unusual in the absence of active variceal bleeding. Based on the pancytopenia, the etiology of the anemia was likely hypersplenism in the setting of portal hypertension. It is possible that there was a brief episode of hemolytic anemia at presentation, however a true autoimmune hemolytic anemia is highly unlikely based on the repeat negative Coombs test and the ease of crossmatching the patient's RBCs. In addition, there may have been chronic microscopic blood loss due to the portal hypertensive gastropathy, however it was not severe, as the patient did not have hematemesis or guaiac positive stools.

Third, the finding of positivity for all three autoantibodies (ANA, anti‐actin, anti‐LKM) is rare. There are two types of AIH based on the positive autoantibody: type 1 is ANA and/or anti‐actin antibody positive, and type 2 is anti‐LKM‐1 and/or anti‐liver cytosolic antibody positive.^4^, ^5^ This patient had evidence for both type 1 and type 2 AIH, however this overlap has not been previously reported.^6^ The abundance of autoantibody positivity begs the question of an underlying genetic immune dysregulation association, and future evaluation is planned.

Finally, the use of tacrolimus in the setting of pancytopenia and borderline liver failure proved to be an effective therapy to aid in the induction and maintenance of remission. Remission is typically induced with corticosteroids and maintained with azathioprine.^7^ However, the patient's pancytopenia and borderline liver failure prompted the use of second‐line tacrolimus therapy up‐front.^7^ This case highlights several significant considerations for clinicians, including the importance of a timely clinicopathologic diagnosis, the severe anemia presentation secondary to hypersplenism, and the rare finding of triple autoantibody‐positive AIH.

## CONFLICT OF INTEREST STATEMENT

The authors declare no conflict of interest.

## ETHICS STATEMENT

Written consent was obtained from the patient and the patient's guardian to submit the case report.
